# Clinical significance of hepatocyte growth factor/c-Met expression in the assessment of gastric cancer progression

**DOI:** 10.3892/mmr.2015.3205

**Published:** 2015-01-15

**Authors:** EIICHIRO NOGUCHI, NOBORU SAITO, MAKIO KOBAYASHI, SHINGO KAMEOKA

**Affiliations:** 1 Department of Surgery II, Tokyo Women’s Medical University, Tokyo 162-8666, Japan; 2 Department of Pathology I, Tokyo Women’s Medical University, Tokyo 162-8666, Japan

**Keywords:** hepatocyte growth factor, c-Met, gastric cancer prognosis, risk factor

## Abstract

Among the mechanisms that control cancer progression, cell mobility is a significant factor required for cellular liberation from the primary focus and infiltration. Hepatocyte growth factor (HGF) has been shown to facilitate cell mobility. In the present study, the clinical significance of the HGF/c-Met pathway in the assessment of gastric cancer progression was evaluated. From a cohort of patients with gastric cancer who underwent surgical resection between April 1999 and March 2003, 110 subjects were randomly selected. Preoperative serum HGF levels were measured and various pathological factors were analyzed. Furthermore, 50 subjects were randomly selected from within this group and immunohistochemical staining of tissue preparations for HGF and its receptor c-Met were performed. In the infiltrative growth pattern [(INF)α,β vs. INFγ], advanced progression was associated with elevated preoperative serum HGF levels (P<0.001). No correlation was identified between serum HGF levels and immunostaining for HGF or c-Met in the tissue preparations. Immunostaining revealed a significant correlation between c-Met expression and lymphatic vessel invasion (ly0.1 vs. 2.3; P=0.0416), lymph node metastasis (n0.1 vs. 2; P=0.0184) and maximum tumor diameter (≤50 mm vs. >50 mm; P=0.0469). Furthermore, c-Met-positivity was associated with a significant difference in overall survival (P=0.0342), despite stage I and II cases accounting for 82% of the total cohort (41 of 50 cases). These results suggested that the expression of the HGF/c-Met pathway in gastric cancer may be a potential predictive factor for disease progression.

## Introduction

Among the mechanisms that mediate cancer progression, cell mobility is a significant factor necessary for liberation from the primary focus and infiltration. Various cell growth factors (1–5), including epidermal growth factor, transforming growth factor β (6,7) and hepatocyte growth factor (HGF) (8), are known to facilitate cell mobility.

HGF, which was first isolated and cloned by Nakamura *et al* (9–12), performs various biological activities in cells, including stimulation of cell growth, promotion of migration, induction of morphogenesis and anti-apoptotic activities, via the c-Met receptor, which is a transmembrane protein containing a tyrosine kinase domain (13–15). The involvement of HGF in the infiltration/metastasis of cancer cells was first suggested in 1991, in a study in which the scatter factor, isolated as a fibroblast-derived bioactive factor with cell stimulatory activities in various cultured epithelial and cancer cells, was found to share an identical structure to that of the HGF molecule (16,17). The functions of HGF were further elucidated by *in vitro* and *in vivo* analyses using various types of cancer cell (18,19). Activation of the HGF/c-Met pathway leads to simultaneous activation of multiple signal transduction pathways that promote the infiltration of cancer cells and is considered to underlie the potent infiltrative/stimulatory effect of HGF (20–25). Genetic mutations of the c-Met receptor have been reported in various cancer types, including papillary renal (20–21), hepatic (22), gastric (23) and pulmonary cancer (24,25), and the overexpression of c-Met has also been reported in numerous cancer tissues (26). Therefore, if the c-Met receptor is present in cancer cells, HGF antagonists should be able to inhibit multiple signal transduction pathways that lead to cancer cell infiltration, thereby exerting potential anti-cancer effects (27).

In a previous study by our group, an association between elevated pre-operative serum HGF levels and advanced disease stages in colon cancer was identified, mainly regarding the depth of tumor invasion into the wall and liver metastasis, which suggested the expression of the HGF/c-Met pathway as a potential predictive factor of colon cancer progression (8). In the present study, serological and immunohistological analyses were conducted in order to evaluate the clinical significance of the expression of the HGF/c-Met pathway in assessing the stage of gastric cancer progression.

## Materials and methods

### Patients

Subjects (n=110) were randomly selected from a cohort of patients with gastric cancer who underwent surgical resection at the Department of Surgery II, Tokyo Women’s Medical University (Tokyo, Japan) between April 1999 and March 2003. Verbal consent was obtained from all patients upon hospitalization and written consent was obtained on the inpatient treatment plan. The study was conducted in 2005 in accordance with the ethical guidelines established by the updated Declaration of Helsinki and Tokyo Women’s Medical University. Preoperative serum HGF levels in these subjects were measured and various pathological factors were analyzed. For 50 of these patients, immunohistochemical staining of tissue preparations for HGF and c-Met was additionally performed in order to analyze various factors identified in serological analysis.

The subjects comprised 83 males and 27 females aged 29–84 years [mean ± standard deviation (SD), 62.8±9.9 years]. The tissue samples were histologically classified as follows: Four as papillary adenocarcinoma, 51 as tubular adenocarcinoma (25 as well-differentiated and 26 as moderately differentiated), 45 as poorly differentiated adenocarcinoma, six as signet-ring cell carcinoma and three as mucinous adenocarcinoma. The histological classification of invasion depth was as follows: Mucosa (m) in 28 patients, submucosa (sm) in 31 patients, muscularis propria (mp) in 11 patients, subserosa (ss) in 18 patients and serosa (se) in 22 patients. The stage classification was IA in 55 patients, IB in 18 patients, II in 16 patients, IIIA in nine patients, IIIB in six patients and IV in six patients (Table I). Data obtained from 200 healthy individuals were used as the control. Healthy individuals comprised patients undergoing surgery for benign diseases, including inguinal hernia or hemorrhoid, and healthy volunteers. Classification of infiltrative growth pattern (INF) was performed according to the General Rules for the Gastric Cancer Society, by the Japanese Research Society for Gastric Cancer, which is based on the Union for International Cancer Control criteria (28).

The 50 subjects that were subjected to immunostaining comprised 38 males and 12 females, with a mean age of 61.8±10.6 years (range, 29–81 years). The tissue samples were histologically classified as follows: One as papillary adenocarcinoma, 23 as tubular adenocarcinoma (12 well-differentiated and 11 moderately differentiated), 20 as poorly differentiated adenocarcinoma, five as signet-ring cell carcinoma and one as mucinous adenocarcinoma. The histological classification of invasion depth was as follows: m in 13 patients, sm in 15 patients, mp in five patients, ss in eight patients and se in nine patients. The stage classification was IA in 25 patients, IB in seven patients, II in nine patients, IIIA in four patients, IIIB in two patients and IV in three patients (Table II).

### Serological analysis

Serum was obtained by centrifugation of venous blood collected prior to surgery at 1,000–2,000 × g for 10 min, which was stored frozen at −80°C and thawed at the time of measurement. HGF levels were measured using a two-step sandwich HGF ELISA kit (Otsuka, Tokyo, Japan), which included the antibodies and o-Phenylenediamine substrate solution, according to the manufacturer’s instructions. In the first reaction, 50 μl phosphate-buffered saline (PBS; Wako Pure Chemical Industries, Ltd, Osaka, Japan) and 50 μl sample were added to each well of a microtiter plate, which was sealed and incubated at room temperature for 1 h with agitation. Following removal of the reaction mixture, the plate was washed five times with wash buffer (Wako Pure Chemical Industries, Ltd). Subsequently, 100 μl/well rabbit polyclonal anti-HGF primary antibody was added for the second reaction and incubated for 1 h at room temperature. Following aspiration and washing five times, 100 μl/well of the horseradish peroxidase-conjugated goat anti-rabbit immunoglobulin G secondary antibody was added for the third reaction and incubated for 1 h at room temperature. Following aspiration and washing five times, 100 μl/well o-Phenylenediamine substrate solution was added. Following incubation at room temperature for 10 min, the reaction was stopped by adding 100 μl of stop solution. Absorbance was measured at 420 nm using a microplate reader (SpectraMax Plus 384; Molecular Devices, Sunnyvale, CA, USA), and HGF levels were determined using a standard curve.

### Immunohistological analysis

HGF: Following deparaffinization with petroleum benzene (Kanto Chemical Co., Inc., Tokyo, Japan) of the 20% formalin-fixed (Wako Pure Chemical Industries, Ltd) paraffin-embedded (Junsei Chemical Co., Ltd, Tokyo, Japan) sections (4 μm), which included the innermost tumor portion of each gastric cancer primary focus, the sections were immersed in PBS and exposed to microwaves at 95°C for 15 min to activate the antigens. Subsequently, the tissue sections were treated with 3% H_2_O_2_ (Sankyo Kagaku Yakuhin Co., Ltd, Kanagawa, Japan) for 20 min to remove the intrinsic peroxidase activity and immunohistochemical staining was performed using the avidin-biotin-peroxidase complex (ABC) method. Following dilution of the reaction with normal horse serum at room temperature for 10 min, rabbit polyclonal anti-human HGF antibody (dilution, 1:20; IBL Co., Ltd, Gunma, Japan) was used as the primary antibody and incubation was continued at room temperature for 60 min. This was followed by reaction with a biotin-conjugated anti-mouse immunoglobulin G secondary antibody (DAKO Japan, Kyoto, Japan) at room temperature for 30 min and reaction with the ABC reagent (DAKO, Glostrup, Denmark) at room temperature for 30 min. The color was developed by addition of 20% 3,3′-diaminobenzidine tetrahydrochloride (Dojindo Laboratories, Kumamoto, Japan), the nuclei were stained with hematoxylin (Merck Millipore KGaA, Darmstadt, Germany) and the sections were dehydrated.

c-Met: c-Met was assayed in a similar manner to HGF, except that the antigen was activated by autoclaving at 95°C for 15 min and a rabbit polyclonal anti-human c-Met primary antibody (dilution, 1:20; IBL Co., Ltd.) was allowed to react at room temperature for 1 h.

**Figure 1:**
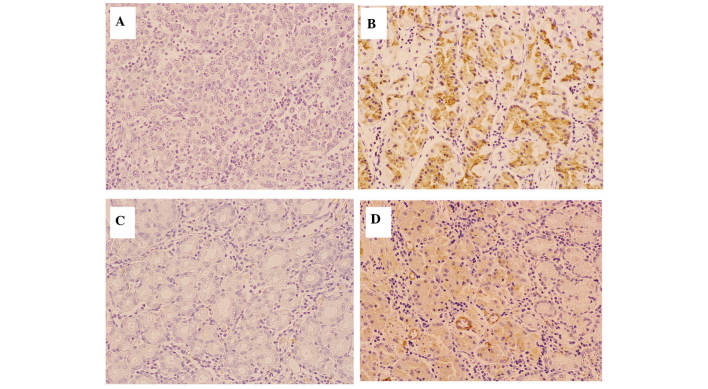
Immunostaining of HGF and c-Met. Microscopic examination was conducted on the tip of the tumor, particularly the innermost part. By observing three fields at 200× magnification, the results were classified as positive if the ratio of stained cancer cells was >25%, as analyzed by comparison. (A) Negative HGF staining. (B) Positive HGF staining. (C) Negative c-Met staining. (D) Positive c-Met staining. HGF, hepatocyte growth factor.

Microscopic examination of HGF and c-Met was performed on the tip of the tumor, particularly the innermost section. Three fields of each section were observed at 200× magnification using a BHS/System Living microscope (Olympus Corp., Tokyo, Japan) and the results were classified as positive when the ratio of stained cancer cells was >25%, according to previous studies that were analyzed for comparison (Fig. 1) (8,29–31).

### Statistical analysis

JMP version 9.0.2 statistical software (SAS Institute, Inc., Cary, NC, USA) was used for statistical analyses. Values are presented as the mean ± SD. The Mann-Whitney *U* test was used to compare differences between two independent groups. Cumulative survival rates were calculated using the Kaplan-Meier method and distributions were identified using the log-rank test. P<0.05 was considered to indicate a statistically significant difference between values.

The terminology used in this report is in accordance with the General Rules of the Gastric Cancer Society by the Japanese Research Society for Gastric Cancer (28).

## Results

### Serological analysis of HGF

Significant differences were detected in preoperative HGF levels between the gastric cancer and control groups (391.0±68.4 vs. 193.3±52.0 pg/ml, respectively; P<0.0001). There was no correlation between preoperative serum HGF levels and patient age or gender. The results of analyses to identify correlations between serum HGF levels and clinicopathological factors are shown in Table I. Advanced progression in the INFα/β vs. INFγ was correlated with elevated preoperative serum HGF levels (P<0.001). Although there was no significant difference in tumor diameter, invasion depth or lymphatic vessel invasion (ly), preoperative serum HGF levels increased as the disease progressed. In patients with peritoneal dissemination, serum HGF levels were frequently increased.

### Immunohistological analysis

**Figure 2:**
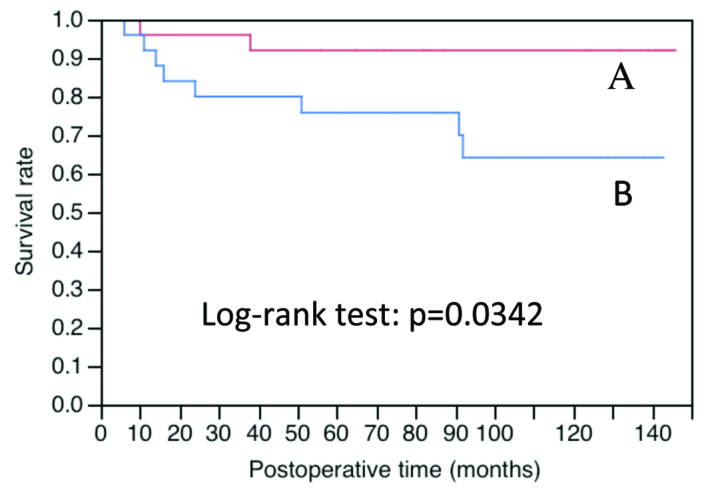
c-Met immunostaining and OS of subjects. OS of c-Met immunopositive cases (n=25) was significantly lower than that of c-Met immunonegative cases (n=25); log-rank test, P=0.0342. (A) Negative c-Met immunostaining. (B) Positive c-Met immunostaining. OS, overall survival.

Of the 50 cases analyzed, 36 (72%) were HGF-positive and 14 (28%) were HGF-negative, whereas 25 (50%) were c-Met-positive and 25 (50%) were c-Met-negative. No correlation was found between serum HGF levels in either staining. There was no correlation between the pathological factors analyzed and HGF levels, whereas a significant correlation was found between c-Met, which is a receptor of HGF, and lymphatic vessel invasion (ly0.1 vs. 2.3; P=0.0416), lymph node metastasis (n0.1 vs. 2; P=0.0184) and maximum tumor diameter (≤50 mm vs. >50 mm; P=0.0469) (Table II). The overall survival (OS) was significantly lower in c-Met-positive cases than that in c-Met-negative cases (P=0.0342; Fig. 2 and Table III).

## Discussion

Cell growth factors, including HGF, constitute a significant group of molecules that regulate cell proliferation, migration and apoptosis in the dynamic organization of cell populations during embryogenesis, organogenesis and regeneration. Numerous factors amongst these additionally promote cell migration. It has been previously reported that HGF has the most potent effect on the promotion of cancer cell infiltration, the cell migration associated with the degradation of extracellular matrix components, including the basement membrane and collagen (9–16). Therefore, activation of the HGF/c-Met pathway results in the simultaneous activation of multiple signal transduction pathways that promote cancer cell infiltration. Antagonists of the HGF/c-Met pathway represent potential anti-cancer agents to inhibit cancer infiltration and metastasis, and therefore, the development of such antagonists is currently underway (27).

In the present study, serological and immunohistological analyses of the expression of the HGF/c-Met pathway in gastric cancer were performed in order to establish its clinical significance in the assessment of disease progression. To the best of our knowledge, no previous studies analyzing serum HGF levels and immunostaining for HGF and c-Met simultaneously with pathological factors were available in the literature.

Although elevated serum HGF levels in patients with gastric cancer had been previously reported (32–35), the present study aimed to determine whether this factor may be used in the assessment of disease progression. The results indicated that pre-operative serum HGF levels were significantly higher in patients with gastric cancer than those in the control group (P<0.0001), and that high HGF levels above the cut-off value (297.3 pg/ml; mean in the control+2 SD) were observed in 93.75% of patients, similar to that reported previously. However, the correlation between HGF levels and disease stage previously reported by Wu *et al* (32) and Han *et al* (33) was not observed in the present study, the results of which were similar to those reported by Taniguchi *et al* (34).

Conversely, advanced progression in the infiltrating growth pattern (INFα/β vs. INFγ) was significantly correlated with high preoperative serum HGF levels (P<0.001). Although this effect may be associated with the involvement of HGF in the infiltrating growth of cancer cells, this factor could not be evaluated because, to the best of our knowledge, no other study on infiltrating growth patterns was available in the literature.

HGF levels were not significantly correlated with certain parameters, including tumor diameter, invasion depth and ly factors; however, preoperative serum HGF levels were elevated as the disease progressed. Regarding the association between HGF levels and invasion depth (pT factor), Niki *et al* (35) identified a significant difference between pT1 and pT2–4 tumors.

Although a significant difference in HGF levels was not detected in patients with peritoneal dissemination, there was a tendency towards high HGF levels among these patients.

Subjects for the present study were selected randomly; therefore no patient with liver metastasis was included. Niki *et al* (35) reported a significant elevation in serum HGF levels in patients diagnosed with liver metastasis, whereas Taniguchi *et al* (34) reported that there was no significant difference in serum HGF levels in patients with relapse independent of liver metastasis. Therefore, the preoperative serum HGF levels in patients with gastric cancer represent a potential predictive factor for disease progression, as observed in colon cancer (6).

In the present study, no correlation was identified between serum HGF levels and immunostaining for HGF or c-Met in tissue preparations; this was potentially due to the complex paracrine and autocrine mechanisms of HGF in cancer cells (36,37). Therefore, the significance of HGF expression in the microenvironment surrounding tumors requires further investigation.

Although there was no correlation between pathological factors and immunostaining for HGF, a significant correlation was identified between c-Met, which is a receptor of HGF, and lymphatic vessel invasion (ly0.1 vs. 2.3, P=0.0416), lymph node metastasis (n0.1 vs. 2, P=0.0184) and maximum tumor diameter (<50 mm vs. >50 mm, P=0.0469). Correlations between immunostaining for c-Met and various pathological factors, particularly invasion depth and disease stage, have been reported in previous studies (38–46). In the present study, cases were selected randomly for immunostaining analysis, as for serological analysis. It was demonstrated that 41 (82%) of the 50 cases analyzed were stage I or II, and 28 (56%) had an invasion depth of m or sm, indicating that the majority of the cohort comprised relatively early stage cancer cases. Only three (6%) cases that were Peritoneum dissemination-factor-positive were stage IV. These results likely explain the absence of statistically significant differences between immunostaining and invasion depth or disease stage.

However, in the present study, which included numerous relatively early cancer cases, the OS of c-Met immunostaining-positive cases was significantly lower than that of negative cases (P=0.0342), indicating that c-Met positivity may be a prognostic factor for gastric cancer.

In chemotherapy for unresectable recurrent gastric cancer, the efficacy of trastuzumab was demonstrated in HER2-positive cases, which subsequently led to the use of personalized drug treatments with molecularly targeted drugs (47). Rilotumumab, which is a fully human monoclonal antibody against HGF and a ligand of the c-Met receptor, suppresses c-Met downstream signaling (47). In pre-clinical models, rilotumumab was shown to inhibit tumor progression in a HGF/c-Met- dependent manner, and its tolerability was verified in early clinical trials (48,49). If future phase II/III trials are implemented under clinical trial designs that allow sufficient verification of the potential of c-Met expression as a biomarker to aid the identification of cases in which rilotumumab is effective, a field of c-Met-positive gastric cancer may be established, similarly to that of HER2-positive gastric cancer. Therefore, further basic studies regarding c-Met expression are required, particularly to improve quality control in immunostaining.

In conclusion, the results of the present study revealed that elevated pre-operative serum HGF levels were indicative of invasive growth of tumor foci, categorized as IFNγ, and characterized by high-grade tumors with an unclear border between the tumor and the surrounding tissue. c-Met-positive immunostaining indicated a tumor with a large diameter, advanced lymphatic vessel invasion and a high degree of lymph node metastasis, and may therefore be a factor indicating poor prognosis. Based on the results described above, the expression of the HGF/c-Met pathway in gastric cancer is a potential predictive factor for disease progression, as previously established for colon cancer.

## Figures and Tables

**Table I:** Clinicopathological factors and serum HGF.

Factor	n	Serum HGF (pg/ml)	P-value
Gastric cancer	110	391.02±68.44	<0.0001
Control	200	193.30±52.00	
Stage^a^			
IA	55	381.21±65.92	NS
IB	18	414.41±82.48	
II	16	378.28±47.01	
IIIA	9	404.64±65.95	
IIIB	6	402.38±93.44	
IV	6	412.94±71.45	
Depth			
m	28	378.89±58.81	NS
sm	31	384.87±69.89	
mp	11	386.97±98.72	
ss	18	403.36±54.88	
s	22	407.04±71.75	
INF^a^			
α	22	369.65±68.93	<0.001 (α.β vs. γ)
β	37	367.34±59.68	
γ	44	418.42±67.72	
Histological type			
well	25	387.23±54.56	NS
mod	26	379.56±80.35	
poor	45	399.25±70.48	
sig	6	404.95±74.10	
muc	3	361.36±77.65	
pap	4	396.12±54.23	
Macroscopic type			
0	59	382.03±64.39	NS
0-advanced	9	364.13±68.61	
1	4	426.32±66.53	
2	16	404.70±87.33	
3	10	402.00±43.63	
4	11	423.53±72.43	
5	1	335.54±0.000	
Lymphatic invasion			
ly0	48	384.71±63.69	NS
ly1	37	391.22±70.67	
ly2	21	398.01±76.63	
ly3	4	428.13±68.74	
Venous invasion			
v0	89	387.92±67.32	NS
v1	20	404.99±75.03	
v2	1	387.52±0.000	
Lymph node metastasis			
n0	78	391.17±70.37	NS
n1	17	382.79±58.94	
n2	13	394.60±68.08	
n3	0		
n4	2	431.73±116.00	NS
Peritoneal dissemination			
p0	105	389.37±68.19	NS
p1	5	425.58±71.99	
Tumor size, mm			
≤–70	97	384.64±66.22	NS
>70	10	428.09±61.82	

a Classification of INF was performed according to the General Rules for the Gastric Cancer Society by the Japanese Research Society for Gastric Cancer. Values are expressed as the mean ± standard deviation.

HGF, hepatocyte growth factor; NS, not significant; m, mucosa; sm, submucosa; mp, muscularis propria; ss, subserosa; s, serosa; INF, infiltrative growth pattern; INFα, expansive type (tumor margin is clear); INFβ, intermediate type; INFγ, invasive type (tumor margin is unclear); well, well-differentiated tubular adenocarcinoma; mod, moderately differentiated tubular adenocarcinoma; poor, poorly differentiated adenocarcinoma; sig, signet-ring cell carcinoma; muc, mucinous adenocarcinoma; pap, papillary adenocarcinoma.

**Table II:** Correlation between HGF/c-Met overexpression and clinicopathological factors.

			HGF expression			c-Met expression		
				
Factor	n	subtotal	(−)	(+)	P-value	(−)	(+)	P-value
All	50		14	36		25	25	
Gender								
Male	38							
Female	12							
Stage								
IA	25	41	12	29	NS	23	18	NS
IB	7							
II	9							
IIIA	4	9	2	7		2	7	
IIIB	2							
IV	3							
Depth								
m	13	13	5	8	NS	9	4	NS
sm	15	37	9	28		16	21	
mp	5							
ss	8							
se	9							
INF								
α	10	32	10	22	NS	16	16	NS
β	22							
γ	17	17	3	14		8	9	
Histological type								
well	12	23	5	18	NS	11	12	NS
mod	11							
poor	20	25	8	17		13	12	
sig	5							
muc	1							
pap	1							
Lymphatic invasion								
ly0	27	43	12	31	NS	24	19	0.0416
ly1	16							
ly2	4	7	2	5		1	6	
ly3	3							
Venous invasion								
v0	43	43	11	32	NS	23	20	NS
v1	7	7	3	4		2	5	
v2	0							
Lymph node metastasis								
n0	34	45	12	33	NS	25	20	0.0184
n1	11							
n2	5	5	2	3		0	5	
n3	0							
n4	0							
Peritoneal dissemination								
p0	47	47	14	33	NS	24	23	NS
p1	3	3	0	3	NS	1	2	NS
Tumor size (mm)								
≤50	38	38	12	26	NS	22	16	0.0469
>50	12	12	2	10		3	9	
Serum HGF (pg/ml)								
≤400	36	36	10	26	NS	17	19	NS
>400	14	14	4	10		8	6	

Mean age (standard deviation) of subjects, 61.8 (10.6) years. HGF, hepatocyte growth factor; SD, standard deviation; NS, not significant; m, mucosa; sm, submucosa; mp, muscularis propria; ss, subserosa; s, serosa; INF, infiltrative growth pattern; INFα, expansive type (tumor margin is clear); INFβ, intermediate type; INFγ, invasive type (tumor margin is unclear); well, well-differentiated tubular adenocarcinoma; mod, moderately differentiated tubular adenocarcinoma; poor, poorly differentiated adenocarcinoma; sig, signet-ring cell carcinoma; muc, mucinous adenocarcinoma; pap, papillary adenocarcinoma.

**Table III:** Five-year survival rate and P-value for overall survival.

Clinicopathological factor	n	Five-year survival rate	P-value
Peritoneal dissemination			
p0	47	0.893	<0.0001 (p0 vs. 1)
p1	3	0.000	
Stage			
I/II	41	0.975	<0.0001 (I/II vs. III/IV)
III/IV	9	0.222	
Tumor size (mm)			
<50	38	0.920	0.0001 (<50 vs. >50)
>50	12	0.583	
Venous invasion			
v0	43	0.906	0.0011 (v0 vs. 1/2)
v1/2	7	0.429	
Lymphatic invasion			
ly0/1	43	0.906	0.0017 (ly0/1 vs. 2/3)
ly2/3	7	0.429	
Lymph node metastasis			
n0/1	45	0.888	0.0056 (n0/1 vs. 2)
n2	5	0.400	
Infiltrative growth pattern			
IFNα/β	32	0.937	0.0083 (IFNα/β vs. γ)
IFNγ	17	0.647	
c-Met expression			
(−)	25	0.920	0.0342 [(−) vs. (+)]
(+)	25	0.758	
serum HGF (pg/ml)			
<400	36	0.887	0.0558 (<400 vs. >400)
>400	14	0.714	
Histological type			
well/mod	23	0.920	0.1793 (well/mod vs. por/sig)
poor/sig	25	0.756	
Depth			
m	13	0.917	0.2649 (m vs. sm/mp/ss/se)
sm/mp/ss/se	37	0.811	
HGF expression			
(−)	14	0.929	0.5385 [(−) vs. (+)]
(+)	36	0.806	

HGF, hepatocyte growth factor; m, mucosa; sm, submucosa; mp, muscularis propria; ss, subserosa; s, serosa; INF, infiltrative growth pattern; INFα, expansive type (tumor margin is clear); INFβ, intermediate type; INFγ, invasive type (tumor margin is unclear); well, well-differentiated tubular adenocarcinoma; mod, moderately differentiated tubular adenocarcinoma; poor, poorly differentiated adenocarcinoma; sig, signet-ring cell carcinoma; muc, mucinous adenocarcinoma; pap, papillary adenocarcinoma.
